# Biomarkers of cardiovascular risk across phenotypes of osteoarthritis

**DOI:** 10.1186/s41927-019-0081-8

**Published:** 2019-08-08

**Authors:** S. A. Provan, S. Rollefstad, E. Ikdahl, A. Mathiessen, I. J. Berg, I. Eeg, I. B. Wilkinson, C. M. McEniery, T. K. Kvien, H. B. Hammer, N. Østerås, I. K. Haugen, A. G. Semb

**Affiliations:** 1 Department of Rheumatology, Oslo, Norway; 2 Preventive Cardio-Rheuma Clinic, Department of Rheumatology, Diakonhjemmet Hospital, Oslo, Norway; 3 Division of Experimental Medicine and Immunotherapeutics, Addenbrooke’s Hospital, University of Cambridge, Cambridge, UK; 4 National Resource Centre for rehabilitation in Rheumatology. Department of Rheumatology, Diakonhjemmet Hospital, Oslo, Norway

**Keywords:** Osteoarthritis, Phenotypes, Ultrasonography, Cardiovascular disease, Arterial stiffness, Central pressure augmentation, Ankle-brachial index

## Abstract

**Background:**

The objective of this study was to explore the associations between ultrasonographic and radiographic joint scores and levels of arterial CVD risk markers in patients with osteoarthritis (OA). Secondly, to compare the levels of arterial CVD risk markers between OA phenotypes and controls.

**Method:**

The “Musculoskeletal pain in Ullensaker” Study (MUST) invited residents of Ullensaker municipality with self-reported OA to a medical examination. OA was defined according to the American College of Rheumatology (ACR) criteria and phenotyped based on joint distribution. Joints of the hands, hips and knees were examined by ultrasonography and conventional radiography, and scored for osteosteophytes. Hands were also scored for inflammation by grey scale (GS) synovitis and power Doppler (PD) signal. Control populations were a cohort of inhabitants of Oslo (OCP), and for external validation, a UK community-based register (UKPC).

Pulse pressure augmentation index (AIx) and pulse wave velocity (PWV) were measured using the Sphygmocor apparatus (Atcor®). Ankel-brachial index (ABI) was estimated in a subset of patients. In separate adjusted regression models we explored the associations between ultrasonography and radiograph joint scores and AIx, PWV and ABI. CVD risk markers were also compared between phenotypes of OA and controls in adjusted analyses.

**Results:**

Three hundred and sixty six persons with OA were included (mean age (range); 63.0 (42.0–75.0)), (females (%); 264 (72)). Of these, 155 (42.3%) had isolated hand OA, 111 (30.3%) had isolated lower limb OA and 100 (27.3%) had generalized OA. 108 persons were included in the OCP and 963 persons in the UKPC; (mean age (range); OCP: 57.2 (40.4–70.4), UKPC: 63.9 (40.0–75.0), females (%); OCP: 47 (43.5), UKPC: 543 (56.4%). Hand osteophytes were associated with AIx while GS and PD scores were not related to CVD risk markers. All OA phenotypes had higher levels of AIx compared to OCP in adjusted analyses. External validation against UKPC confirmed these findings.

**Conclusions:**

Hand osteophytes might be related to higher risk of CVD. People with OA had higher augmented central pressure compared to controls.

Words 330.

**Electronic supplementary material:**

The online version of this article (10.1186/s41927-019-0081-8) contains supplementary material, which is available to authorized users.

## Background

Osteoarthritis (OA) is a prevalent disease, characterized by derangement of the whole joint. The classification criteria are clinical and radiographic [1–3]. The prevalence of OA in an urban European population aged ≥55 years has been estimated, OA of the distal inter-phalangeal joint was most common with 33% being afflicted, knee OA was present in 15% while hip OA was found in 6% [4]. Ultrasonography is useful for the detection of osteophytes and current inflammation at the joint level, the latter visualised by grey scale (GS) synovitis and power Doppler (PD) signal.

Cardiovascular disease (CVD) accounts for over 60% of deaths in OA populations [5], and there is evidence of increased all-cause, and cardiovascular disease (CVD) related, mortality in OA [4, 6, 7]. The increased risk of CVD is often attributed to the established covariation between OA and established risk factors of CVD such as decreased physical activity, increased body mass index (BMI) and prevalent use of non-steroid anti-inflammatory drugs (NSAIDs) [5, 8]. Vascular pathology as a result of low level inflammation at the joint level is however a causal pathway that is also debated [6]. Arterial stiffening is a pathophysiological step in CVD development, and a predictor of CVD mortality [9]. The gold standard of arterial stiffness evaluation is pulse wave velocity (PWV) and the relative risk (RR) for CV mortality increases by 15% (95% CI 9–21) for each 1 m/s increase in PWV [10]. The augmentation index (AIx) is a measure of central pressure augmentation and also an independent risk factor of CVD events and mortality, and the relative risk of CV events is estimated to increase by 32% (95% CI 9–59) for each 10% increase in AIx [11]. Ankle-brachial index (ABI) is a validated marker of CVD risk [12]. At cut-off of 0.9 is most frequently used for ABI but the mortality rate falls linearly with increasing ABI until a floor is reached at an ABI of approximately 1.1. There is some indication of an increased mortality rate if ABI > 1.4. An ABI < 0.9 is associated with an increased risk of CVD, with an OR for CV event between 1.3 and 4.2 reported in a review of 9 papers [12].

The objectives of this study were to investigate the association between radiographic and ultrasonographic joint scores in OA and CVD risk markers. Secondly, to compare CVD risk markers between phenotypes of OA and population controls, adjusting for possible confounders.

## Methods

### The musculoskeletal pain in Ullensaker study (MUST)

Residents of Ullensaker municipality (Norway), aged 42–80 years, with self-reported OA were invited to a comprehensive data-collection including medical examination (details previously published [13]). Individuals aged between 42 and 75 were selected for this study. The MUST study was approved by the Norwegian Regional Committee for Medical and Health Research Ethics South East (reference numbers 2009/812 and 2009/1703) and the participant gave their written informed consent according to the Declaration of Helsinki prior to inclusion.

### Oslo community controls (OCP)

A random sample of 329 adult inhabitants of Oslo (Norway), aged 20–70, were selected by Statistics Norway, and individuals without an inflammatory joint disease were invited to a cardiovascular risk assessment at Diakonhjemmet Hospital. ABI, radiography and US joint examinations were not performed in this cohort [14]. In total 134 (41%) consented. The OCP was approved by the Norwegian Regional Committee for Medical and Health Research Ethics South East (reference numbers 2009/1703) and the participants gave their written informed consent according to the Declaration

### UK population controls (UKPC)

For external validation of our findings we used anonymised data from The Anglo-Cardiff Collaborative Trial. This was a community population-based study of ≈ 12,000 individuals drawn from General Practice lists or open access cardiovascular risk assessment units in East-Anglia and Wales, United Kingdom. The response rate was 85%. From this cohort a random sample of 963 cases, aged 40–75 years, without self-reported inflammatory rheumatic disease or OA was included.

### Joint examinations (MUST only)

In MUST, hand, knee and hip joints were examined and radiographs of hands, knees and hips taken as previously described [13]. Radiographic OA severity was scored according to the Kellgren-Lawrence (KL) scale (grade 0–4) for hands (distal and proximal interphalangeal, metacarphalangeal and first carpometacarpal joints), hip and knee joints. OA was defined according to the American College of Rheumatology (ACR) classification criteria [1–3]. For hand OA, only clinical variables are included in the criterion, whereas both clinical and radiographic variables are included in the criteria for hip and knee OA. When radiographs were missing, the clinical ACR criterion for knee OA was used if sufficient data was available. The cut-offs for joint space narrowing (JSN) and osteophytes in the ACR criterion for hip joints were grade ≥ 1 according to the Osteoarthritis Research Society International atlas [15]. Knee osteophytes were defined as KL grade ≥ 2. Persons with hip or knee prosthesis were classified as having OA in the respective joint. Participants were categorised according to the following OA phenotypes based on the fulfilment of the ACR criteria: 1) Isolated hand OA, 2) Isolated OA in lower limbs (i.e. knee and/or hip OA), 3) Generalized OA, defined as OA in both hands and lower limbs.

The protocol for ultrasonography and conventional radiographs of bilateral hands, knees and hips has been previously described [13]. Osteophytes GS and PD were semi-quantitatively scored on 0–3 scales (0 = none, 1 = minor, 2 = moderate and 3 = major presence of US pathology). Sum scores for ultrasound defined osteophytes were calculated for hands (range 0–90) and hips/knees (range 0–30), GS and PD in the hands (range 0–90) and GS hips/knees (range 0–4). KL sum scores were calculated for the hands (range 0–120) and hips/knees (range 0–16).

### Biomarkers of CVD risk

Pulse wave analyses (PWV and AIx) were performed using the Sphygmocor apparatus (Atcor®) by trained personnel using the same methodology for all cohorts (MUST, OCP and UKPC) [13, 16]. ABI was measured in a standardized fashion. To obtain the ABI, the systolic pressure was estimated by a Sonotrax - Pocket Doppler Vascular - 8 MHz probe in the posterior tibial and dorsalis pedis arteries in both legs and in the brachial artery in one arm. The highest pressure recorded in either artery was recorded as the measurement for that side. The lowest of the distal pressures (ankle) measured either at the right or the left side was then divided by the brachial systolic pressure [12].

In MUST recordings of AIx were available in 258 (70.5%), PWV in 257 (70.2%) and ABI in 174 (47.5%) persons. Missing PWA recordings were related to logistical limitations of the study, unavailability of the PWA investigator and/or poor data quality according to predefined criteria. Due to time limitations ABI was recorded in every other patient as previously described [13]. In OPC, AIx was available in 104 (96.2%) and PWV in 97 (89.8%) of the participants. All UKPC participants had available AIx and PWV. ABI was not assessed in OCP and UKPC.

### Covariates

All cohorts had information concerning age, gender body mass index (BMI) measured in kg/m^2^, C-reactive protein (CRP) measured as mg/L and smoking status, dichotomized as daily smoker vs. non-daily smoker or non-smoker. MUST and OCP also had information on level of education, dichotomized as college or university education vs. high school or lower education, use of non-steroidal anti-inflammatory drugs (daily vs. non-daily or never), physical functioning by the Modified Health Assessment Questionnaire (MHAQ) and self-reported exercise habits (> = once a week vs. < once a week) [13].

### Statistics

This study was designed as a cross-sectional case-control study, with two separate control populations. Variables were visually examined for normality, and kurtosis and skewness were calculated. Baseline demographics were compared using chi-square tests, independent samples Student’s t-test or Mann-Whitney U-test as appropriate.

In linear regression models we examined the associations between ABI, AIx, PWV (dependent variables) and the severity of OA using ultrasonography and radiographic and sum scores. The estimated marginal means of AIx and PWV were estimated and compared between OA phenotypes, OCP and UKPC. ABI was compared between OA phenotypes. The associations between AIx, PWV, ABI and all covariates were tested in age and gender adjusted models, andco-variates associated with the dependent outcome with a p < = 0.01 were included in the final models. AIx models were also adjusted for heart rate and height. PWV models were adjusted for heart rate.

Missing data was not imputed, but a comparison of demographics between participants with available vs. missing PWA and ABI was performed. Cooks distances were calculated and outliers examined.

## Result

**Fig 1:**
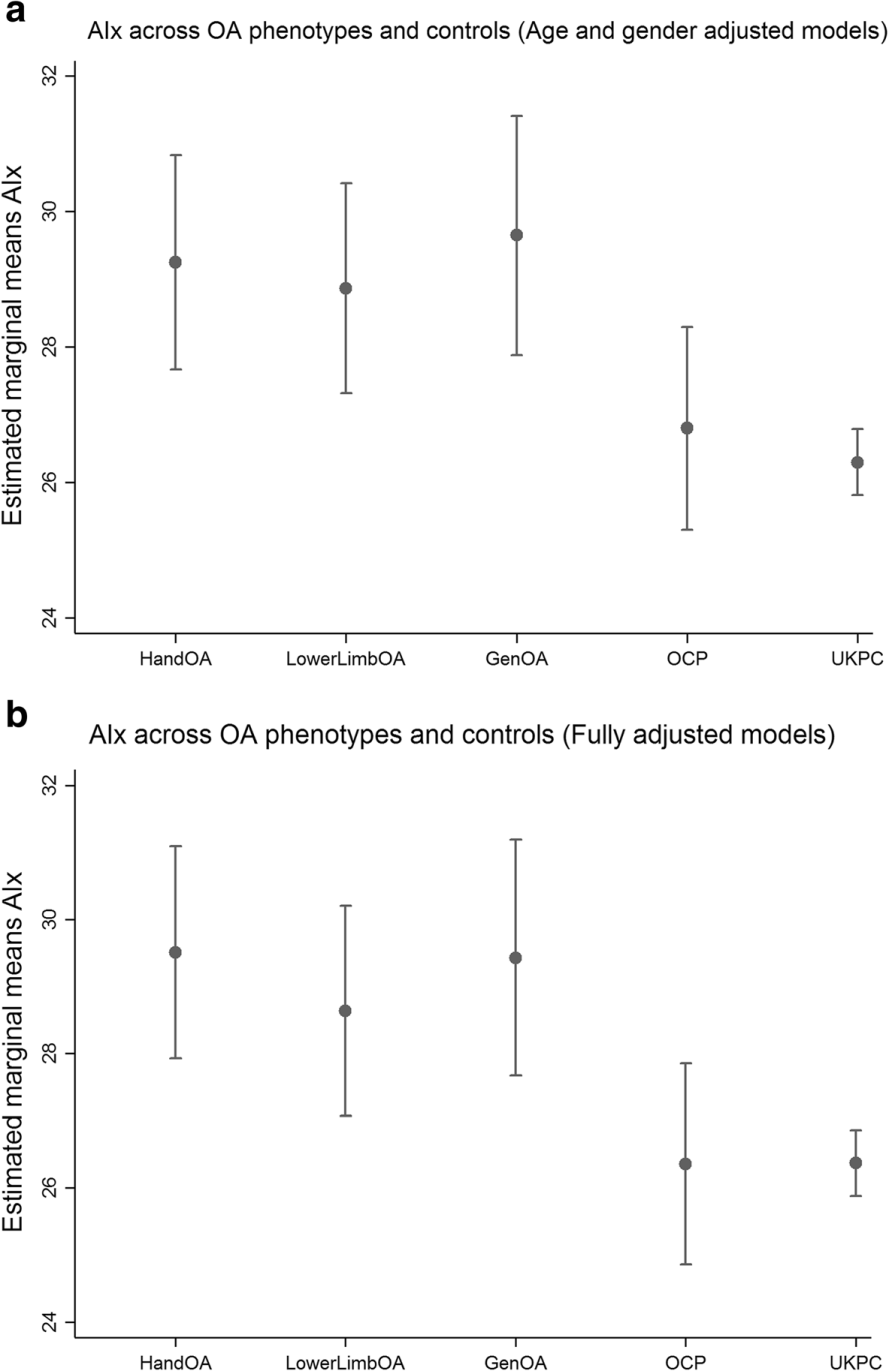
**a** AIx across OA phenotypes and controls (Age and gender adjusted modesl) Vertical axis: Estimated marginal means AIx. **b** AIx across OA phenotypes and controls (Fully adjusted models) Vertical axis: Estimated marginal means AIx. AIx; augmentation index OA; osteoarthritis Gen; general, OCP; Oslo community population, UKPC; United Kingdom Community population. Footnotes a HandOA vs OCP *p* = 0.03, vs. UKPC *p* = 0.001, LowerLimbOA vs OCP *p* = 0.06, vs. UKPC *p* = 0.002, GenOA vs. OCP *p* = 0.02, vs. UKPC *p* < 0.001, HandOA vs LowerLimbOA *p* = 0.73, HandOA vs GenOA *p* = 0.74, GenOA vs LowerLimbOA *p* = 0.51. b HandOA vs OCP *p* = 0.004, vs. UKPC p < 0.001, LowerLimbOA vs OCP *p* = 0.04, vs. UKPC *p* = 0.007, GenOA vs. OCP *p* = 0.009, vs. UKPC p = 0.001, HandOA vs LowerLimbOA *p* = 0.44, HandOA vs GenOA *p* = 0.95, GenOA vs LowerLimbOA *p* = 0.50

We examined 630 persons with self-reported OA and of these 366 persons with OA were identified. Of the 366 persons; 155 (42.3%) had isolated hand OA according to the ACR criteria, 111 (30.3%) had isolated lower limb OA, and 100 (27.3%) had generalized OA. Details of the selection procedure are presented in Fig. 1. Demographic and clinical characteristics of the OA phenotypes of the persons included in the MUST are presented in Table 1, and for OCP and UKPC in Additional file 1: Table S1. Persons with generalized OA were significantly older than persons with isolated hand OA, while persons with isolated lower limb OA were significantly less likely to be female.

**Table 1:** Demographic and clinical characteristics of the OA phenotypes in MUST

	Number with variable	Hand OA *n* = 154	Lower-Limb OA *n* = 111	General OA *n* = 100	p Hand vs Lower-limb	p Lower-limb vs. Gen. OA	p Hand vs Gen. OA
Age, mean (range)	366	62.1 (42.0–75.0)	62.9 (45.0–75.0)	64.3 (47.0–75.0)	0.40	0.18	0.02
Female gender, n (%)	366	126 (81.3)	62 (55.9)	76 (76.0)	< 0.001	0.002	0.31
Smoking daily, n (%)	363	18 (11.7)	13 (11.9)	12 (12.0)	0.95	0.99	0.94
Higher education, n (%)	359	37 (24.5)	41 (37.3)	26 (26.5)	0.03	0.10	0.72
BMI kg/m^2,^ mean (SD)	356	25.8 (4.3)	28.0 (4.7)	28.1 (4.7)	< 0.001	0.77	< 0.001
CRP mg/L, mean (SD)	364	2.2 (3.5)	2.6 (4.4)	2.9 (3.8)	0.44	0.64	0.17
Use of NSAIDs daily n (%)	364	27 (17.4)	20 (18.2)	20 (20.2)	0.87	0.71	0.58
Heart rate, beats/min (SD)	258	64.4 (10.4)	63.7 (9.8)	65.4 (10.7)	0.63	0.29	0.56
MHAQ	366	1.20 (1.16–1.25)	1.20 (1.15–1.25)	1.26 (1.19–1.32)	0.98	0.19	0.16
Regular exercise n (%)	365	137 (77.4)	101 (91.0)	87 (87.9)	0.50	0.46	0.90
AIx,% mean (SD	258	32.3 (8.5)	29.0 (8.9)	31.7 (8.6)	0.01	0.05	0.67
PWV m/s, mean (SD)	257	8.65 (2.0)	8.7 (1.9)	9.1 (2.0)	0.61	0.25	0.11
ABI	174	1.2 (0.1)	1.2 (0.1)	1.2 (0.2)	0.02	0.46	0.14

Unadjusted bivariate models. The Chi square, independent samples Student T-test or independent samples

Mann-Whitney U-test were used as appropriate

*N* number, *BMI* body mass index, *CRP* C-reactive protein, *NSAIDs* non-steroidal anti-inflammatory drugs, *AIx* augmentation index, *PWV* pulse wave velocity, *BP* blood pressure. *MUST-OA* persons in MUST with osteoarthritis, *OCP* Oslo community controls, *UKPC* UK population controls

Severity of structural OA changes in the hands (KL sum score and US-defined osteophytes) was significantly associated with AIx. However, neither synovitis (hand GS and PD, and lower limb GS), nor osteophytes in the lower limbs (hip and knee KL sum scores and US-defined osteophyte hip and knee sum score) were significantly associated with the CVD risk markers (Table 2).

**Table 2:** The association between CVD vascular biomarkers, demographics, covariates and OA radiographic and ultrasonographic variables

	CVD risk markers (dependent variables)		
	AIx β (95% CI)	PWV β (95% CI)	ABI β (95% CI)
Patients in MUST, OPC and UKPC			Only MUST
Age years, mean (range)	0.27 (0.21–0.33)**	0.12 (0.11–0.13)**	− 0.00 (− 0.01–0.00)
Female gender, n (%)	6.38 (5.26–7.49)*	− 0.22 (− 0.42- -0.02)*	− 0.10 (− 0.15—0.06)**
Smoking daily, n (%)	3.60 (2.19–5.01)**	0.15 (− 0.20–0.50)	− 0.05 (− 0.11–0.02)
Higher education, n (%)	−0.06 (− 1.77–1.65)	− 0.03 (− 0.39–0.32)	0.02 (− 0.02–0.06)
BMI kg/m^2,^ mean (SD)	−0.05 (− 0.14–0.04)	0.06 (0.04–0.09)**	0.00 (− 0.00–0.01)
CRP mg/L, mean (SD)	− 0.02 (− 0.10–0.06)	0.03 (0.01–0.05)*	0.00 (− 0.00–0.00)
Use of NSAIDs	1.00 (− 1.11–3.11)	0.14 (− 0.31–0.58)	− 0.02 (− 0.07–0.03)
MHAQ	1.30 (−1.79–4.38)	− 0.16 (− 0.80–0.48)	−0.00 (− 0.68–0.07)
Regular exercise n (%)	−1.59 (− 3.63–0.45)	−0.10 (0.53–0.34)	−0.02 (0.08–0.05)
Heart rate (beats/min)	−0.45 (− 048- -0.41)**	0.04 (0.03–0.05)**	−0.00 (− 0.00–0.00)
Patients in MUST only			Only MUST
Radiographic osteophytes Hand KL	0.07 (0.01–0.13)*	0.00 (−0.01–0.02)	0.00 (− 0.00–0.00)
Radiographic osteophytes Lower limb	− 0.19 (− 0.66–0.29)	−0.04 (− 0.15–0.07)	0.01 (0.00–0.02)*
US PD Hand	0.41 (− 1.45–2.27)	0.05 (− 0.36–0.46)	−0.01(− 0.05–0.03)
US GS Hand	0.28 (− 0.06–0.63)	−0.04 (− 0.12–0.03)	0.00 (− 0.00–0.01)
US GS Lower limb	−0.84 (− 2.64–0.96)	−0.06 (− 0.47–0.34)	0.01 (− 0.03–0.05)
US osteophytes Hand	0.09 (0.02–0.16)*	−0.00 (− 0.00–0.00)	0.00 (− 0.00–0.00)
US osteophytes Lower limb	0.07 (−1.39–1.54)	−0.19 (− 0.52–0.13)	0.02 (− 0.02–0.05)

Dependent variables in separated models. *N* number, *BMI* body mass index, *CRP* C-reactive protein, *NSAIDs* non-steroidal anti-inflammatory drugs, *AIx* augmentation index, *PWV* pulse wave velocity. *BP* blood pressure. *MUST-OA* persons in MUST with osteoarthritis, *OCP* Oslo community controls, *UKPC* UK population controls, *AIx* augmentation index, *PWV* pulse wave velocity, *ABI* ankle-brachial index

All models adjusted for age and gender. Linear regression models *β* beta coefficient, *CI* confidence interval, *n* number, *KL* Kellgren Lawrence, *PD* power Doppler *GS* grey scale *p < 0.05**p < 0.00

**Fig. 2:**
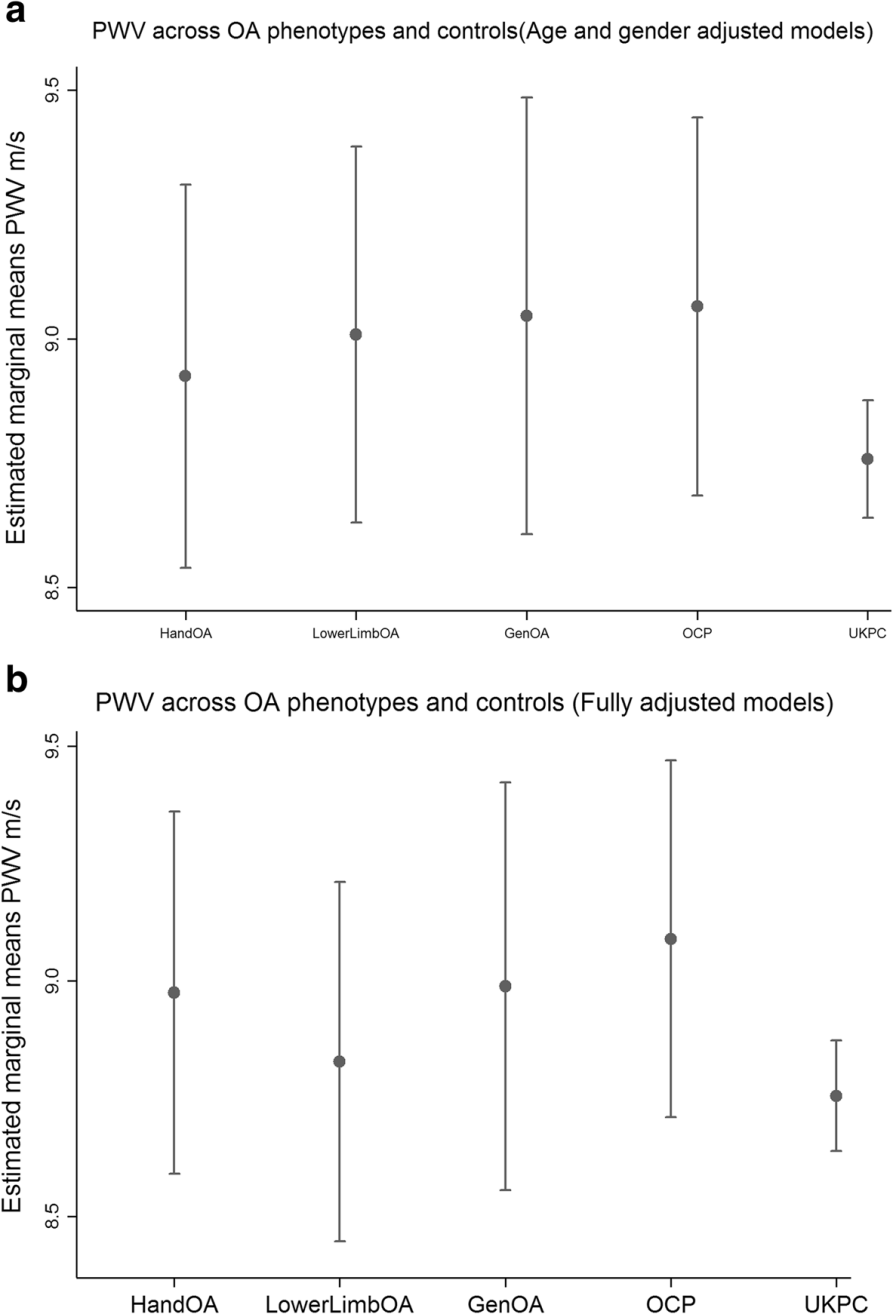
**a** PWV across OA phenotypes and controls (Age and gender adjusted models). Vertical axis: Estimated marginal means PWV m/s. **b** PWV across OA phenotypes and controls (Fully adjusted models). Vertical axis: Estimated marginal means PWV m/s. PWV; Pulse wave velocity OA; osteoarthritis Gen; general, OCP; Oslo community population, UKPC; United Kingdom Community population. Footnote **a** HandOA vs OCP *p* = 0.61, vs. UKPC *p* = 0.42, LowerLimbOA vs OCP *p* = 0.83, vs. UKPC *p* = 0.22, GenOA vs. OCP p = 0.95, vs. UKPC *p* = 0.21, HandOA vs LowerLimbOA *p* = 0.76, HandOA vs GenOA *p* = 0.68, GenOA vs LowerLimbOA *p* = 0.90. **b** HandOA vs OCP p = 0.68, vs. UKPC *p* = 0.29, LowerLimbOA vs OCP *p* = 0.34, vs. UKPC *p* = 0.72, GenOA vs. OCP p = 0.73, vs. UKPC *p* = 0.31, HandOA vs LowerLimbOA *p* = 0.59HandOA vs GenOA *p* = 0.96, GenOA vs LowerLimbOA *p* = 0.58

**Fig. 3:**
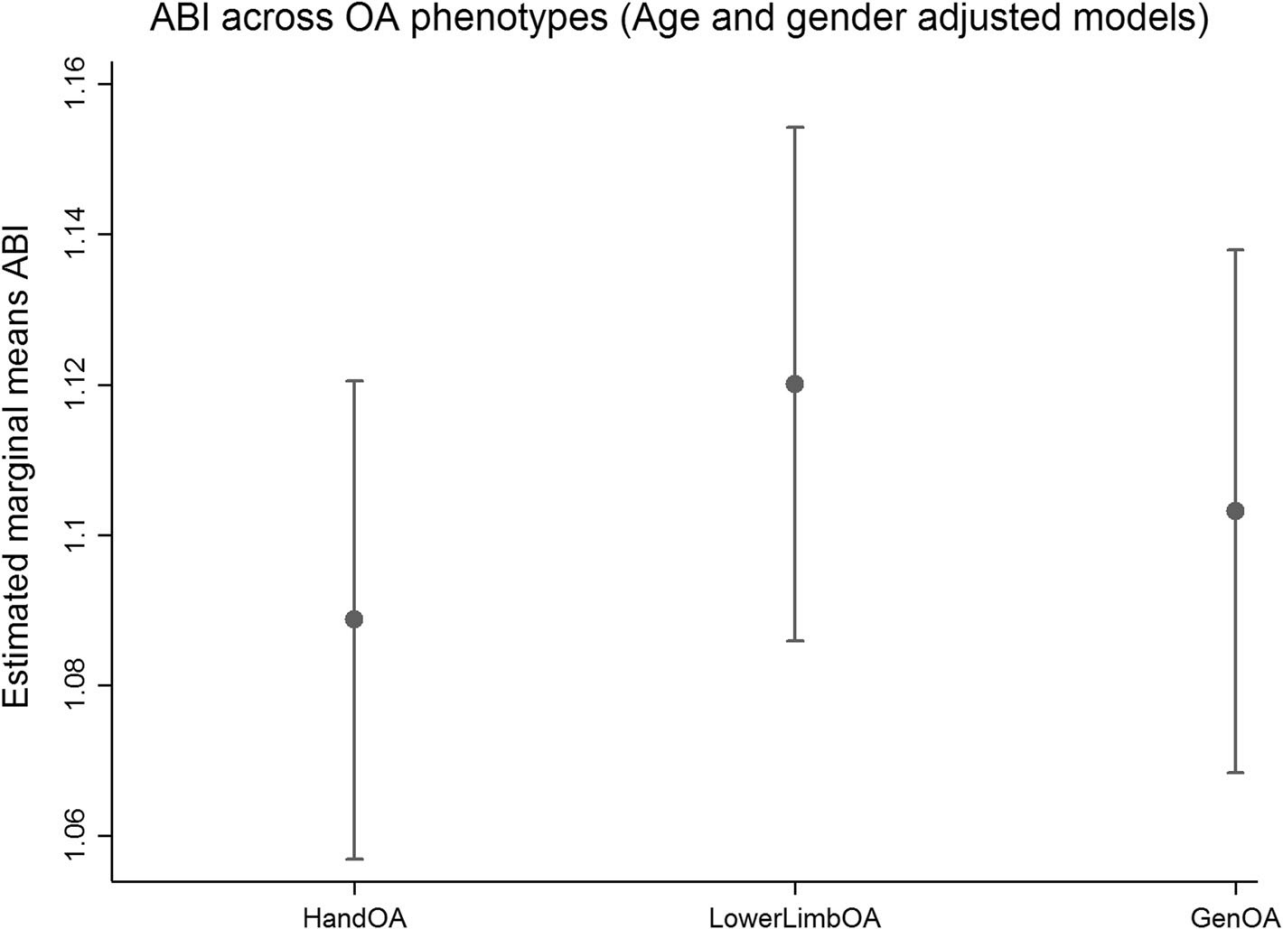
Footnote both figures. ABI; Ankle brachial index Pulse wave velocity OA; osteoarthritis Gen; general. HandOA vs LowerLimbOA *p* = 0.19, HandOA vs GenOA *p* = 0.55, GenOA vs LowerLimbOA *p* = 0.05

Participants with OA had higher AIx compared to OCP and UKPC, regardless of phenotype (Fig. 1a). The final multivariate model for AIx also adjusted for smoking, but this did not significantly change the main effect (Fig. 1b). PWV and ABI were not increased in patient with OA compared to OCP and UKPC (Fig. 2a and 3a). The final model for PWV adjusted for BMI, but this did not alter the lack of association between OA and PWV (Fig. 2b). None of the covariates were significantly associated to ABI in the final model, except for gender. There were no significant differences in AIx, PWV or ABI between the OA phenotypes.

Comparisons of persons in MUST with vs. without CVD risk markers are presented in the Additional file 1: Table S2. More females than males had missing AIx and PWV.

## Discussion

In this study structural hand OA severity was associated with AIx, but there were no association between level of inflammation in the hands, measured by GS and PD, and other CVD risk markers. Furthermore, participants with OA had higher levels of AIx compared to OCP controls, and this finding was confirmed by external validation against the UKPC.

To our knowledge, we are the first group to investigate the relationship between US joint scores and CVD risk markers in people with OA. Vascular pathology has been hypothesised to be both a risk factor for OA initiation and development, and a possible consequence of the changes in extra-cellular matrix and cartilage seen in OA. Thus, an examination of the association between vascular CVD-risk markers and OA imaging scores is of interest [6, 17]. The AIx is considered a more precise estimation of central hemodynamics than brachial pressure, and has also been shown to be a predictor of CVD that is independent of brachial pressure [11].

In our study CVD risk, measured by increased levels of AIx, was not associated with acute inflammation at the joint level graded by US, measured by GS and PD, or with CRP. However, AIx was associated with structural OA features, which may be considered the cumulative result of joint inflammation [18]. In addition, participants with symptomatic hand OA, fulfilling the ACR criteria, had higher AIx compared to controls. The AGES study from Iceland also found that carotid plaques and coronary calcium scores were associated with the severity of hand OA judged by photographs of the hands [7, 19]. Haugen et al. found a higher risk of coronary heart disease in persons with symptomatic hand OA, but not for persons with non-symptomatic radiographic hand OA in the Framingham study [20].

The severity of lower-limb OA was not significantly related to CVD risk markers in this study, although patients with lower limb and general OA did have higher levels of AIx, compared to controls in our study. There have been disparate findings on a possible connection between lower limb OA joint destruction and CVD risk. Goldsmith et al. reported that bone marrow lesions in knee OA were not related to the levels of a panel of vascular CVD-risk markers [17]. Belen et al. however, found that aortic stiffness was correlated to the KL score in knee OA [21], and Tootsi et al concluded that radiographic OA grade was associated with PWV in a cohort of patients with end stage knee and hip OA [22].

Large systematic reviews have demonstrated an excess risk of CVD in persons with OA [5, 6], our study indicates that central augmented pressure, measured by AIx is increased in patients with OA in general, and particularly in patients with structural hand OA evident as osteophytes. One possible explanation for the increased AIx in structural hand OA is that the hands are organs at the end of a vascular loop, and that change in vascular resistance as a consequence of structural joint and vascular pathology may increase the wave reflection in the arteries, thus increasing the augmented pressure. In contrast, acute inflammation is associated with vascular dilatation and thus reduced AIx [14].

The clinical relevance of this study is that it identifies an association between structural hand OA and increased central augmented pressure, which may in the future lead to patients with OA, and structural hand OA in particular, receiving anti-hypertensive treatment at an earlier stage. In patients with lower limb or general OA the association between CVD risk and structural joint pathology was not evident, but presence of OA was associated with an increased level of central pressure. Although a possible linkage between joint vascular pathology, also in the joints of the lower limb, and risk of CVD cannot be negated, possible confounders and mediators must be considered. A strength of our study was that we had the opportunity to examine the association between CVD-risk markers and BMI, CRP, smoking, education, physical functioning, physical activity and use of NSAIDs, and adjust the models when warranted, but these covariates did not alter the associations.

Other strengths of this study were the comprehensive data-collection and the heterogeneity of OA phenotypes in the MUST cohort. A limitation is however that this is a cross-sectional study and causal relationships cannot be explored. A further limitation is that the OCP control cohort was not designed as a comparator for MUST and although the participants in both MUST and OCP come from the same region of Norway, the cohorts do not match perfectly with regards to gender distribution and age range. We have adjusted for these differences in our analyses, and we also chose to use the large UKPC for external validation of our findings. We also acknowledge that approximately 15% of participants had not performed PWA, and that participants without PWA were more likely to be female compared to participant where PWA was available. Despite the gender difference we believe that the PWA was missing at random due to logistical challenges of this large study.

## Conclusion

Structural hand OA severity might be related to higher risk of CVD. People with OA had higher augmented central blood pressure compared to controls.

## Additional files

Additional file 1: **Table S1.** Bivariate comparisons between participants with OA and UK population controls. **Table S2.** Comparison between participants with and without AIx, PWV and ABI. (DOCX 40 kb)

## Data Availability

Please contact author for data requests.

## Abbreviations

ABI: ankle-brachial index
ACR: American College of Rheumatology
AIx: Augmentation index
BMI: Body mass index
CVD: Cardiovascular disease
GS: Grey scale
KL: Kellgren-Lawrence
MHAQ: Modified Health Assement Questionnaire
MUST: Musculoskeletar pain in Ullensaker
NSAID: Non-steroidal anti-inflammatory drugs
OA: Osteoarthritis
OCP: Oslo control population
PD: Power Doppler
PWV: Pulse wave velocity
UKPC: UK population controls
