# Supplementary material for: Biomarkers of cardiovascular risk across phenotypes of osteoarthritis
Source: BMC Rheumatol. 2019 Aug 8;3:33. doi: 10.1186/s41927-019-0081-8 (PMC6686275; doi:10.1186/s41927-019-0081-8)
Supplement: Supplementary file 1 — Table S1. Bivariate comparisons between participants with OA and UK population controls. Table S2. Comparison between participants with and without AIx, PWV and ABI. (DOCX 40 kb) [file 41927_2019_81_MOESM1_ESM.docx]

|  | OCP n= 108 | UKPC  n= 963 | HandOA  vs. OCP p | Lower limb OA vs. OCP p | Gen OA vs. OCP p | HandOA vs. UKPC p | Lower limb OA vs. UKPC p | Gen. OA vs. UKPC p |
| --- | --- | --- | --- | --- | --- | --- | --- | --- |
| Age, mean (range) | 57.2  (40.4-70.4) | 63.9  (40.0-75.0) | <0.001 | <0.001 | <0.001 | 0.003 | 0.17 | 0.54 |
| Female gender, n (%) | 47 (43.5) | 543 (56.4) | <0.001 | 0.07 | <0.001 | <0.001 | 0.92 | <0.001 |
| Smoking daily, n (%) | 25 (23.2) | 68 (7.2) | 0.01 | 0.03 | 0.04 | 0.05 | 0.08 | 0.09 |
| Higher education, n (%) | 56 (52.3) | - | <0.001 | 0.03 | <0.001 | - | - | - |
| BMI kg/m^2,^ mean (SD) | 25.8 (3.9) | 26.7 (4.5) | 0.97 | <0.001 | 0.001 | 0.02 | 0.007 | 0.003 |
| CRP mg/L, mean (SD) | 3.4 (10.7) | 3.0 (4.8) | 0.22 | 0.49 | 0.66 | 0.06 | 0.44 | 0.83 |
| Use of NSAIDs daily n (%) | 15 (14.0) | - | 0.46 | 0.40 | 0.24 | - | - | - |
| Heart rate, beats/min (SD) | 63.0 (10.2) | 70.3 (11.2) | 0.35 | 0.63 | 0.14 | <0.001 | <0.001 | <0.001 |
| MHAQ | 1.1 (1.0-1.1) | - | <0.001 | <0.001 | <0.001 | - | - | - |
| Regular exercise  n (%) | 61 (58.1) | - | <0.001 | <0.001 | <0.001 | - | - | - |
| AIx,% mean (SD | 25.5 (10.5) | 26.0 (10.2) | <0.001 | 0.01 | 0.001 | <0.001 | 0.006 | <0.001 |
| PWV m/s, mean (SD) | 8.1 (1.9) | 8.9 (2.1) | 0.07 | 0.02 | 0.001 | 0.17 | 0.46 | 0.46 |

Supplementary Table 1 Bivariate comparisons between participants with OA and UK population controls

Unadjusted bivariate models. The Chi square, independent samples Student T-test or independent samples

Mann-Whitney U-test were used as appropriate.

N; number, BMI; body mass index, CRP; C-reactive protein, NSAIDs; non-steroidal anti-inflammatory drugs, AIx; augmentation index, PWV; pulse wave velocity. BP; blood pressure. MUST-OA; persons in MUST with osteoarthritis, OCP; Oslo community controls, UKPC; UK population controls

|  | Missing AIx n=112 | AIx available n=1326 | Missing PWV n= 120 | PWV available n= 1318 | Missing ABI n=190 | ABI available  n=176 |
| --- | --- | --- | --- | --- | --- | --- |
| Age years (SD) | 62.5 (7.7) | 63.2 (7.2) | 62.4 (7.5) | 63.2 (7.2) | 62.9 (7.6) | 63.0 (7.6) |
| Female gender n(%) | 86 (76.8) | 768 (58.0)** | 84 (70) | 770 (58.5)* | 135 (71.1) | 129 (73.3) |

Supplementary table 2 Comparison between participants with and without AIx, PWV and ABI

*p<0.05 ** p<0.001

SD; standard deviation, AIx; augmentation index, PWV; pulse wave velocity, ABI; Ankle–brachial index
